# Mechanisms associated with the trajectory of depressive and anxiety symptoms: A linear mixed-effects model during the COVID-19 Pandemic

**DOI:** 10.1007/s12144-022-02732-9

**Published:** 2022-02-04

**Authors:** Omid V. Ebrahimi, Asle Hoffart, Sverre Urnes Johnson

**Affiliations:** 1 Modum Bad Psychiatric Hospital, Vikersund, Norway; 2 Department of Psychology, University of Oslo, Forskningsveien 3A, Harald Schjelderups hus, 0373 Oslo, Norway

**Keywords:** Anxiety, Depression, Symptom trajectories, Mechanisms, COVID-19, Social distancing

## Abstract

With the fluctuations in anxious and depressive symptomatology accompanied by the pandemic crises, studies on the trajectories of these symptom domains are warranted to monitor the development of mental health problems in the population. This pre-registered longitudinal study examines stable factors and mechanistic processes covarying with the trajectory of anxiety and depressive symptoms using linear-mixed effects models in 4936 adults from the pandemic’s onset to four months into the COVID-19 pandemic in Norway. Prevalence estimates of moderate to severe levels of clinically impairing symptoms of anxiety and depression revealed high but reduced occurrence four months into the pandemic where social distancing protocols were substantially lightened in severity, revealing associations between symptoms and viral mitigation protocols after stringent control of plausible confounders. Subgroups at risk at the onset of the pandemic sustained their relative position compared to their counterparts four months into the pandemic, indicating prolonged suffering of these subgroups. Among mechanistic processes, key differences were identified regarding the trajectory of anxiety and depressive symptoms. Physical exercise was associated with long-term but not momentaneous alleviations in anxiety. In contrast, reductions in depressive symptoms were associated with both the simultaneous exertion as well as dose-increases in exercise over time. Increased knowledge about how to best cope with pandemic challenges was associated with greater improvement in depressive but not anxiety symptoms. Reductions in maladaptive coping strategies and negative metacognitive beliefs was substantially associated with greater improvement of both anxious and depressive symptomatology. Mechanistic processes divergently relate to the trajectory of depressive and anxious symptomatology, yielding domain-specific information of utility for preventive and interventive efforts aimed at impeding deleterious symptom levels.

**Supplementary Information:**

The online version contains supplementary material available at 10.1007/s12144-022-02732-9.

## Introduction

The arrival of the COVID-19 pandemic has been accompanied by what scholars term a parallel pandemic consisting of detrimental mental health symptoms (e.g., Yao et al., 2020). This phenomenon has been empirically corroborated in a wide range of studies portraying substantial elevations in anxious and depressive symptomatology since the onset of the pandemic (e.g., Ebrahimi et al., 2021a; Ettman et al., 2020; Salari et al., 2020; Wang et al., 2020). Although these studies have advanced the pandemic mental health literature by uncovering prevalence rates of anxiety and depression in association with their covariates, their implementation of cross-sectional designs preclude insight concerning the trajectories which these symptom domains follow during pandemics and the factors intertwined with such changes across time. Consequently, knowledge remains scarce concerning the mechanistic processes covarying with the drastic changes witnessed in the mental health symptoms of the population. To mirror this scarcity, calls have been made for studies to investigate the trajectories of anxious and depressive symptomatology during the COVID-19 pandemic (Chi et al., 2020; Rossi et al., 2020; Sun et al., 2021).

Several attempts have been made to respond to such calls for research concerning the course of change in symptoms. Presently, findings from available studies inspecting the trajectory of change in anxious and depressive symptomatology have shed light on several important risk factors related to unfavorable symptom development in pandemic periods. A recent study by Fancourt et al. (2021) found multiple demographic factors predictive of the trajectories of depressive and anxiety symptoms, inter alia identifying faster rates of improvement for females as time passed during the first wave of the pandemic. Similarly, other studies have identified key sociodemographic risk factors related to the temporal changes in mental health symptoms, including age, education, and living alone (e.g., Luchetti et al., 2020; Riehm et al., 2021). Although studies on demographic disparities as covariates of the trajectories of anxious and depressive symptomatology are imperative from a population-wide resource-allocative and preventive perspective, the identification of these stable demographic risk factors presents limited actionable utility from a clinical-interventive stance. Accordingly, the preponderance of concurrent trajectorial studies on mental health during the pandemic remain descriptive in nature and restricted to findings connected to stable demographic characteristics. A shift toward mechanisms may remedy this issue, with mechanisms referring to the processes entwined with triggering events and the internal and external reactions to these events, which attribute to the amplification and maintenance of symptoms (e.g., Hoffart & Johnson, 2020; Kazdin, 2009). Knowledge remains scarce concerning actionable mechanistic processes which may be interwoven with the observed changes in anxious and depressive symptomatology during the pandemic, which in turn can provide guidance concerning the adaptation of interventive strategies in mitigating the rise in adverse mental health symptoms. Moreover, as noted by Fancourt et al. (2021), there is a lack of studies investigating the distinct and differential trajectories of anxiety and depression during the pandemic, with several existing studies agglutinating these symptom clusters into a unitary ‘mental distress’ outcome (e.g., Fancourt et al., 2021; Riehm et al., 2021). Improved understanding of mechanisms covarying with the alleviation of mental symptoms has further been argued as important from a public health perspective, with elevations in anxiety having the possibility to foster detrimental behavioral responses during viral outbreaks (e.g., Asmundson & Taylor, 2020).

Pertaining the fluctuations in mental health symptoms, although the pandemic itself and its mitigation protocols are cogent correlates of detrimental symptoms of anxiety and depression, it is likely that their impact on psychopathological symptomatology functions through central mechanistic processes associated with psychopathological symptoms. Concerning changes in the trajectories of depression and anxiety, plausible mechanistic processes may encompass of fluctuations in maladaptive coping strategies, including the preservative processes of worry and rumination, which have previously been associated with symptoms of anxiety and depression cross-sectionally in pandemic settings (e.g., Ebrahimi et al., 2021a; Elhai et al., 2021; Skjerdingstad et al., 2021; Taylor et al., 2020). Moreover, changes in the reliance of other maladaptive strategies (e.g., consumption of alcohol as regulatory strategy) and its covariance with the trajectories of symptoms of anxiety and depression warrants investigation, as the adaptation of such strategies may have flourished for certain individuals in attempting to cope with a novel, and for many, an unprecedented crisis. Indeed, several studies demonstrate that alcohol consumption has increased during the pandemic, with its increased usage detrimentally associated with both depressive and anxious symptomatology in cross-sectional studies (Avery et al., 2020; Jacob et al., 2021; Taylor et al., 2021). Additionally, metacognitive beliefs including positive and negative metacognitions are previously hypothesized transdiagnostic mechanisms related to both depression and anxiety (e.g., Wells, 2009). It remains unclear whether the initial levels and changes in such beliefs during the course of the pandemic may influence the trajectory of anxious and depressive symptomatology. Moreover, changes in pandemic-specific variables such as increases in one’s perception of competence in coping with pandemic challenges may alter the course of depressive and anxious symptomatology, with the pandemic presenting a range of novel situations and challenges requiring adaptation. It is additionally important to investigate the association between mechanisms related to lifestyle and mental health during the pandemic, with changes in factors such as physical activity being of possible relevance to anxious and depressive trajectories. These aforementioned factors all serve as actionable mechanistic processes subjectable to manipulation by pre-existing efficacious treatments. Consequently, the extent to which they are associated with changes in the trajectory of psychopathological symptoms in the general population is a matter of importance to public health, in addition to clinicians and clinical scientists. This holds true both in pandemic and non-pandemic settings, but is of particular concurrent relevance due to the observed elevations in the aforementioned symptom domains during the pandemic. In this light, this study aims to investigate the association between the baseline levels and changes across time in the mechanistic processes including metacognitive beliefs, maladaptive strategies, physical activity, and perceived competence with the trajectory of anxious and depressive symptomatology during the COVID-19 pandemic. These symptom trajectories were followed from the onset of the pandemic in March (T1), a period with intensive pandemic mitigation protocols in place, to July (T2) where these invasive social distancing protocols were substantially lightened in severity.

The present pre-registered study organizes its findings in a threefold manner, starting with presentations of a) changes in prevalence of clinically impairing depressive and anxiety symptoms from the onset of the pandemic (T1) as compared to four months into the pandemic where viral mitigation protocols were lightened (T2), revealing in-risk demographic subgroups along the way. Subsequently, b) the associative link between the trajectories of anxiety and depressive symptoms with the baseline levels of actionable mechanistic processes (e.g., physical activity and maladaptive coping strategies) are presented, providing insight into long-term associations and delayed effects. Importantly, c) changes in these mechanistic processes across time and their association with the trajectory of anxious and depressive symptomatology is presented, yielding insight concerning the mechanisms associated with the maintenance and alleviation of these detrimental mental health symptoms during the pandemic.

## Methods

This pre-registered study is part of the Norwegian COVID-19, Mental Health and Adherence Project. Ethical approval was granted by the Regional Committee for Medical and Health Research Ethics (reference: 125510​). The report is prepared pursuant to the guidelines of the GATHER statement (Stevens et al., 2016), with items in the statement concerning presentation of details such as objectives of the study, funding, the methods and analyses utilized, and the description of inclusion criteria for the study (i.e., eligible participants). The protocol of this investigation was pre-registered prior to collection of data, available at Clinicaltrials.gov (Identifier: NCT04442204). All elements of the submitted study adhere to its pre-registered protocol.

### Design and recruitment

This two-wave longitudinal study of the general adult population investigates the levels of depressive and anxiety symptoms approximately four months into the pandemic outbreak in Norway (T2) as compared to the onset of the pandemic in March (T1). Eligible participants included all individuals aged 18 years and above who were currently living in Norway and accordingly experiencing an identical set of nationally implemented viral mitigation protocols. The first wave of data collection was between March 31 and April 7, 2020 (T1; *N* = 10061), a period with intensive social distancing protocols implemented, such as isolation upon infection, quarantine upon contact with those infected, restrictions of social gatherings, prohibitions of public activities and events, closing of universities and schools, and visitation and domestic travel restrictions. All participants were re-invited to participate in the second wave of data collection (T2) where *N* = 4936 of the 10061 (i.e., 49.06%) subjects responded to the survey which was collected between June 22 and July 13, 2020, a period where the preponderance of social distancing protocols were lightened in severity (e.g., domestic travel restrictions removed, schools re-opened, public activities events up to 200 individuals allowed, and size of social contact group contact increased to 20 individuals). A list of the implemented viral mitigation protocols, also commonly termed non-pharmacological interventions (NPIs), present at the two assessment waves of the study is provided in Supplementary Information 1 in tables S1 and S2, respectively.

To provide the adult population with an equal opportunity to participate in the study, the survey was primarily disseminated using a Facebook Business algorithm to any adult residing in Norway. This algorithm disseminates the survey to a random sample of the proportion of the adult population available on Facebook (i.e., 85% of the entire adult in Norway). Seventy percent of the overall participants included in this study are obtained through this random selection technique. To reach the residual 15% of Norwegian adults not on this platform, the survey was disseminated systematically through national, regional, and local platforms (i.e., newspapers, radio stations, and television) across the entire country.

The study design emphasized controlling for expectation effects as well as impacts of modifications of the viral modification protocols. Correspondingly, all implemented mitigation protocols were a) identical across all regions, b) kept constant for at least two weeks, and c) unmodified throughout both waves of data collection. Expectation effects were controlled for by implementing a stopping rule in the study design which would stop data collection instantaneously if any information concerning modification of viral mitigation protocols were provided. Moreover, the study design involved recruitment of a proportionate number of participants from each region of Norway compared to the population of that region, yielding a geographically representative sample of the adult population. The survey was administered digitally and in a random order to the participants.

### Measures

Participants reported their demographic information including their sex, age, and education level. Participants were further queried about the number of days out of the preceding 14 days that they had socially distanced themselves from peers and public activity related to the pandemic mitigation protocols. Individuals who reported to have socially distanced themselves for at least 10 of the preceding 14 days were coded as having predominantly socially distanced from peers and public activity.

#### Symptoms of anxiety

Anxiety symptoms were measured with the Generalized Anxiety Disorder 7 (GAD-7; Spitzer et al., 2006), consisting of seven items measuring anxiety on a four-point Likert scale (0–3; 0 = Not at all, 3 = Nearly every day), with scores ranging from 0 to 21. Internal consistency was good, with a Cronbach’s *α* of .90. Higher scores indicate greater anxiety severity. A commonly used cut-off for GAD-7 scores includes 8 or above, further validated as the cut-off for determining the presence of clinically impairing (i.e., moderate) levels of anxiety symptoms in Norwegian samples (Johnson et al., 2019).

#### Symptoms of depression

Depressive symptoms were measured with the Patient Health Questionnaire (PHQ-9; Kroenke et al., 2001), consisting of nine items scored on a four-point Likert-scale (0–3; 0 = Not at all, 3 = Nearly every day). Scores range from 0 to 27. The PHQ-9 was selected given its measurement of depressive symptomatology as outlined by the Diagnostic and Statistical Manual of Mental Disorders (DSM; American Psychiatric Association, 2013), its wide-spread use and well-normed cut-offs in the adult population, its high accuracy as a diagnostic screening tool, in addition to calls for its specific use with respect to its validity (e.g., Nature Medicine, 2020). Higher scores on the PHQ-9 indicate greater depression severity, with scores above and including 10 considered as the cut-off revealing clinically impairing (i.e., moderate) levels of depressive symptoms, indicative of a depressive diagnosis with a sensitivity and specificity of 88% (Kroenke et al., 2001). The internal consistency of this scale was good in this sample, with a Cronbach’s *α* of .91.

#### Cognitive Attentional Syndrome Scale-1

The Cognitive Attentional Syndrome Scale-1 (CAS-1; Nordahl & Wells, 2019) is a 16-item scale consisting of three subscales developed to measure metacognitive beliefs and reliance on maladaptive strategies. The first subscale consists of eight items (*α* = .91) and measures the extent of maladaptive coping strategies on a nine-point Likert Scale (0–8; 0 = Never; 8 = All the time). Maladaptive strategies include the deployment of worry and rumination as regulatory strategies to cope with negative thoughts and emotions, in addition to reliance on other regulatory strategies such as the use of alcohol or substances. The scores on this subscale range from 0-64 with higher scores reflecting greater reliance on maladaptive coping strategies.

**Fig. 1:**
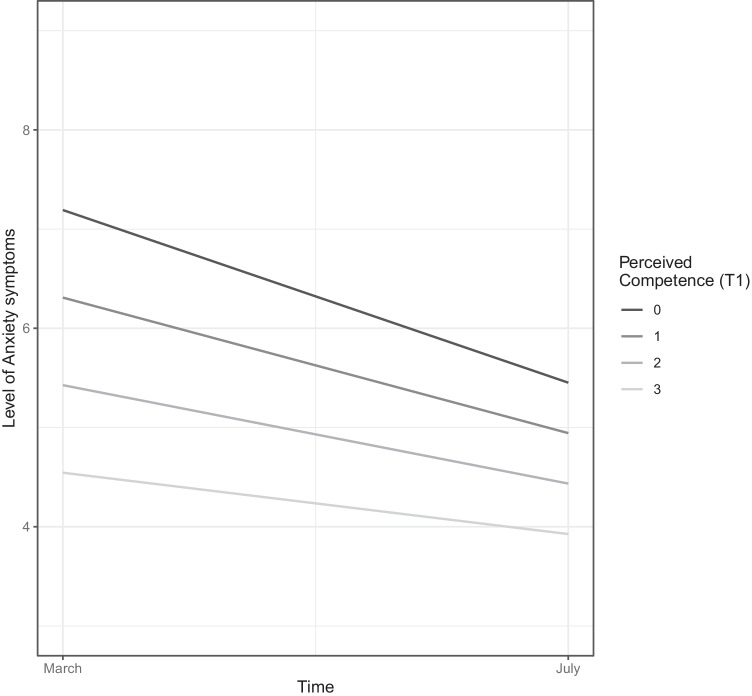
The trajectory of anxiety symptoms as predicted baseline (T1) levels of perceived competence. The figure depicts the association of the variable with the criterion while controlling for all other variables in the model

**Fig. 2:**
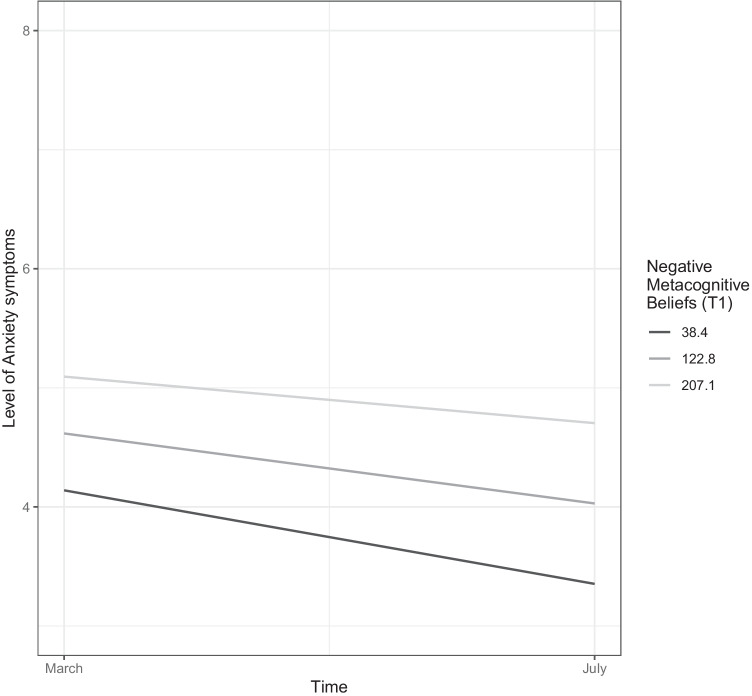
The trajectory of anxiety symptoms as predicted by baseline (T1) levels of negative metacognitions. The figure portrays the association of the variable with the criterion while controlling for all other variables in the model

**Fig. 3:**
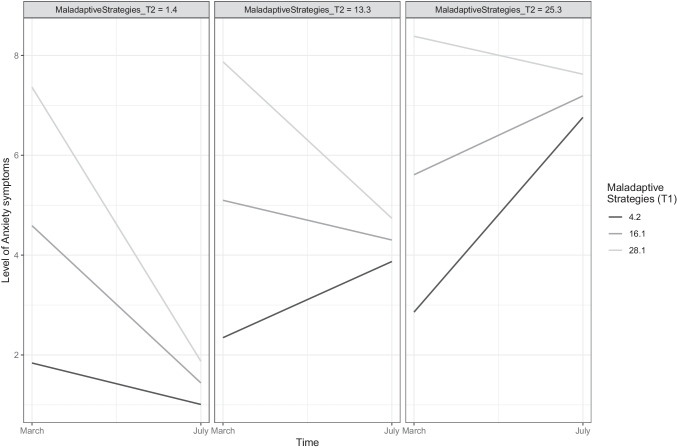
The trajectory of anxiety symptoms as predicted by changes in maladaptive coping strategies from T1 to T2. The figure depicts the association of the variable with the criterion while controlling for all other variables in the model

**Fig. 4:**
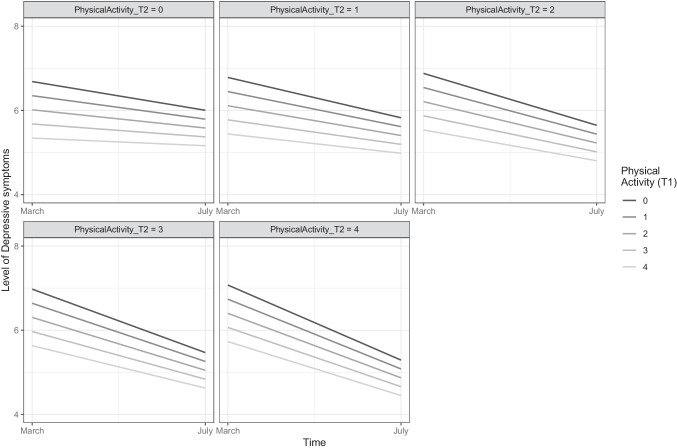
Trajectory of depressive symptoms as predicted by changes in physical activity from T1 to T2. The figure reveals the association of the variable with the criterion while controlling for all other variables in the model

The positive metacognitive beliefs subscale (*α* = .63) consists of four items, concerning positive assumptions related to the process of worry, including items such as “worrying helps me cope”. The final subscale of the CAS-1 concerns negative metacognitive beliefs (4 items, *α* = .71) such as “some thoughts could make me lose my mind”. Both latter subscales are scored on a scale from 0 (“I do not believe this at all”) to 100 (“I’m completely convinced this is true”), yielding scores ranging from 0 to 400, with higher scores reflecting greater presence of positive or negative metacognitive beliefs, respectively. Given the large range of scores on the CAS-1 subscales (e.g., 0 to 400 for positive and negative metacognitive beliefs), the reader interested in clinically relevant sizes of associations between these variables and the criterion variable may multiply the provided regression coefficients by 80 (i.e., the average standard deviation in metacognitive beliefs across both time-points). As standard for continuous measures, candidate values representing prevalent and typical scores in the dataset will be represented in Figures 1, 2, 3 and 4, providing visual aid in tracking symptom changes across time in addition the relative strength of association.

#### Physical activity

Physical activity was operationalized as engagement in an activity lasting for a minimum of 30 minutes and leading to light sweat and/or increased pulse. This operationalization was conducted to distinguish between less intensive movement versus more intensive physical exercise. The extent of physical activity was measured in accordance with its frequency during the past two weeks on a five-point Likert scale (0-4; 0 = Not at all, 4 = More than every other day (i.e., above 8 times).

#### Perceived competence in handling pandemic challenges

The participants perceived competence to deal with challenges related to the pandemic was measured with an item from the Basic Psychological Needs and Frustration Scale (Chen et al., 2015) adapted for the present pandemic context (“I feel confident in my abilities to deal with the challenges related to the pandemic crisis”) on a four-point Likert-scale (0-3; 0 = Not at all; 3 = Nearly every day).

### Statistical analyses

Statistical analyses were performed in R (version 4.0.2) using linear mixed effects-models with the ‘lme4’ package (Bates et al., 2015). Plots are depicted using the ‘ggeffects’ package (Lüdecke, 2018). Descriptive statistics were reported using means and standard deviations, and difference tests between subgroups regarding prevalence of clinically impairing symptoms of anxiety and depression were conducted using chi-squared tests.

Longitudinal surveys involving multiple waves of data collection typically have missing data. Accordingly, linear mixed effects-models were conducted utilizing maximum likelihood estimation, the state-of-art approach in handling missing data (Schafer & Graham, 2002). In preliminary analyses and for each of the two criterion variables (i.e., symptoms of anxiety and depression), the combination of random effects and covariance structure of residuals that provided the best fit for the “empty” model (i.e., the model without fixed predictors except the intercept) was chosen. The Akaike’s Information Criterion (AIC) was used to compare the fit of different models. Models that gave reductions in AIC greater than 2 were considered as superior (Burnham & Anderson, 2004). To test whether symptom levels changed between T1 to T2, symptoms of anxiety and depression were used as dependent variables in their respective analyses, using a model with time as a predictor (T1 = 0; T2 = 1). To a) test the associations between baseline (T1) levels of predictors with the trajectory of depression and anxiety symptoms, in addition to b) testing how changes (i.e., from T1 to T2) in the mechanistic processes were related to changes in depression and anxiety, two models were built (i.e., denoted as Model 1 and Model 2, respectively), both including the demographic variables age, sex, and education levels as control variables and predictors of change in the trajectory of anxious and depressive symptomatology. Specifically, Model 1 includes the baseline (T1; at the onset of the pandemic) levels of negative metacognitive beliefs, positive metacognitive beliefs, maladaptive coping strategies, physical activity, and perceived competence to deal with the pandemic as constant covariates, together with the interactions of these covariates with time. The aforementioned interaction terms in Model 1 inform whether the baseline levels of these covariates predict change in the trajectory of anxiety and depression symptoms. In Model 2, the T2 levels of negative metacognitions, positive metacognitions, maladaptive coping strategies, physical activity, and perceived competence to deal with the pandemic were added as constant covariates, together with their interaction with time. The interaction terms in Model 2 represent tests of whether the change in the covariates from T1 to T2 predict change in depressive and anxious symptomology from T1 to T2, respectively. The inference criteria for the analyses were pre-registered and defined at *p* < .01 with respect to the large sample size and the multiple comparisons conducted in the study.

Several figures are provided to aid interpretation, with two types of figures presented. Figures 1, 2 and Supplementary Figures 1-2 reveal the trajectory of depressive and anxious symptoms as predicted by baseline (T1) levels of mechanistic variables and demographic characteristics. The figure legend portrays the trajectory for anxiety and depressive symptoms for a specific value of the predictor, while controlling for all other predictors (i.e., all other demographic and mechanistic variables) in the model.

Figures 3, 4 and Supplementary Figure 3 illustrate how changes in the mechanistic variables from T1 to T2 (e.g., increasing physical activity levels from T1 to T2) is related to the trajectory of anxious and depressive symptomatology. These two figures depict how going from a particular level at baseline (depicted by the figure legend) to a specific level at T2 (depicted by title line in each of the boxes or sub-figures) on a mechanistic variable is related to change in the trajectory of anxiety and depressive symptoms. These figures also illustrate the association between change in each mechanistic variable and the trajectory of anxiety and depressive symptoms while controlling for all other included variables in the study. To illustrate this, the reader is referred to Figure 4, with the darkest line depicting that increasing physical activity levels at T1 from the value ‘0’ (No activity) to a value of ‘4’ (More than every other day) at T2 is associated with a greater reduction in depressive symptoms than maintaining one’s level of physical activity (maintaining the value ‘0’ at T2). Following the darkest line sequentially (i.e., from left to right) in all subfigures of Figure 4 reveals that greater increase in physical activity from T1 to T2 more substantially reduces depressive symptoms.

In all figures (i.e., 1-4), candidate values representing prevalent and typical scores in the dataset will be presented for continuous variables (i.e., maladaptive coping strategies, negative metacognitions, positive metacognitions, and age), while all levels of the ordinal and categorical variables (i.e., physical activity, perceived competence, and sex) are presented.

## Results

### Sample description

Overall, 4936 individuals participated in the study at T2, *M*_age_ = 38.93, with the demographic details provided in Table 1. The sample consisted of approximately 78-79% females at T1 and T2, respectively. All analyzed subgroups were richly represented in the dataset (e.g., 2184 males at T1 and 1010 males at T2), and sensitivity analyses on the same group of participants (see Ebrahimi et al., 2021a) revealed the sample as accurate and representative for the general adult population following analysis on a) solely the randomly selected proportion of participants, in addition to b) on an adjusted, post-stratified and weighted sample matching the sample characteristics to the population, both of which replicated and revealed indifferent results as the main sample. The recruited participants were further geographically representative of Norway, with the ratio of individuals from each region being proportionate to the population parameter. Additionally, the proportion of pre-existing mental health conditions in this sample was 18.03%, which reflects the lower end of the known rate of psychological disorders in the adult population of Norway, which is between 16.66% to 25.00% (Norwegian Institute of Public Health, 2016). Akin to findings from other nations (e.g., Capasso et al., 2021; Taylor et al., 2021), 22.23% of the participants in the present Norwegian sample reported that they had increased their consumption of alcohol since the onset of the pandemic.

**Table 1:** Demographic characteristics of the participants at both waves of data collection. Data at T1 was collected between March 31 to April 7, 2020. T2 encompassed of the period between June 22 to July 13, 2020

Subgroups	T1: *N* (%)	T2: *N* (%)
**All participants**	10 061	4936
**Age group, years**		
18-30	4706 (46.77%)	1711 (34.66%)
31-44	2849 (28.32%)	1610 (32.62%)
45-64	2142 (21.29%)	1347 (27.29%)
65+	364 (3.62%)	268 (5.43%)
**Sex**		
Female	7851 (78.03%)	3911 (79.23%)
Male	2184 (21.71%)	1010 (20.46%)
Intersex	4 (0.04%)	13 (0.26%)
Transgender	22 (0.22%)	2 (0.04%)
**Self-identifies with biological sex**		
Yes	10010 (99.49%)	4908 (99.43%)
No	51 (0.51%)	28 (0.57%)
**Civil Status**		
Single or divorced	5310 (52.78%)	2337 (47.35%)
Married or in a civil union	4751 (47.22%)	2599 (52.65%)
**Education Level**		
Completed Elementary School	522 (5.19%)	192 (3.89%)
Completed High School	1784 (17.73%)	741 (15.01%)
Currently studying	2111 (20.98%)	779 (15.78%)
University or College Degree	5644 (56.10%)	3224 (65.32%)
**Currently Employed**		
Employed	8140 (80.91%)	3780 (76.58%)
Unemployed	1921 (19.09%)	1156 (23.42%)

### Prevalence of depressive and anxiety symptoms at T2 compared to T1

There was a significant reduction in anxiety (estimate = -.94, *SE* = .05, *t* (5810) = -19.24, *p* < .001) and depressive symptoms (estimate = -.77, *SE* = .06, *t* (5701) = -13.60, *p* < .001) from T1 (i.e., period of strict pandemic protocols) to T2 (i.e., period of predominant discontinuation of pandemic protocols). Overall, 994 out of 4936 participants (20.14%) reported clinically impairing (i.e., moderate to severe) levels of anxiety symptoms at T2 (compared to 27.57% at T1), while 1195 (24.21%) reported impairing levels of depressive symptoms at T2 (compared to 30.78% at T1).

As outlined in Table 2, all previously identified subgroups (i.e., females, individuals that were single or divorced, unemployed subjects, individuals belonging to ethnic minorities and sexual orientation minorities) that reported higher levels of impairing anxious and depressive symptomatology at the onset of the pandemic (T1; Ebrahimi et al., 2021b) maintained their relative position compared to their counterparts four months into the pandemic (T2). Additionally, individuals living alone were identified as having substantially greater risk of experiencing impairing depressive (30.60% vs. 22.30%, *p* < .001) and anxiety symptoms (23.46% versus 19.15%, *p* = .001) compared to their counterparts (i.e., individuals residing with others, including families, partners, or friends).

**Table 2:** Levels of psychiatric symptoms in the general adult population (*N* = 4936) four months into the COVID-19 pandemic. Percentage meeting cut-offs for clinically significant (i.e., moderate to severe) levels of anxious and depressive symptoms are provided. Differences between subgroups are reported using chi-squared tests *χ*^*2*^

	*N*	Mean (SD), Range	Percentage of subgroups meeting diagnostic cut-off (%)	Chi-squared test, *χ*^*2*^
**Symptoms of anxiety (GAD-7)**				
**All participants**	4936	4.66 (4.37), 0-21	20.14%	
**Sex**				*χ*^*2*^ (3, *N* = 4936) = 98.30, *p* < .001
Female	3911	4.91 (4.62), 0-21	21.60%	
Male	1010	3.69 (3.98), 0-21	14.26%	
Intersex	2	5.00 (2.83), 3-7	NA^b^	
Transgender	13	7.31 (5.85), 0-17	38.46%	
**Identification with biological sex**				*χ*^*2*^ (1, *N* = 4936) = 4.067, *p =* .044
Yes	4908	4.65 (4.36), 0-21	20.05%	
No	28	6.32 (4.92), 0-17	35.71%	
**Civil status**				*χ*^*2*^ (1, *N* = 4936) = 50.69, *p* < .001
Single or divorced	2337	5.31 (4.58), 0-21	25.25%	
Married or in a civil partnership	2599	4.08 (4.08), 0-21	15.54%	
**Ethnicity**				*χ*^*2*^ (1, *N* = 4936) = 11.43, *p* < .001
Native	4634	4.61 (4.35), 0-21	19.70%	
Non-native	302	5.51 (4.59), 0-20	26.82%	
**Employment status**				*χ*^*2*^ (1, *N* = 4936) = 56.92, *p* < .001
Employed	3780	4.25 (3.97), 0-21	16.96%	
Unemployed	1156	6.00 (5.25), 0-21	30.54%	
**Predominantly socially distanced**^**a**^				*χ*^*2*^ (1, *N* = 4936) = 36.21, *p* < .001
Yes	3892	4.92 (4.45), 0-21	21.92%	*χ2 (1, N = 4936) = 36.21, p < .001*
No	1044	3.71 (3.89), 0-21	13.51%	
**Symptoms of depression (PHQ-9)**				
**All participants**	4936	6.23 (5.66), 0–27	24.21%	
**Sex**				*χ*^*2*^ (3, *N* = 4936) = 41.41, *p* < .001
Female	3911	6.90 (5.65), 0-27	25.67%	
Male	1010	5.52 (5.55), 0-27	17.92%	
Intersex	2	7.07 (7.07), 1-11	NA^b^	
Transgender	13	11.62 (6.35), 0-19	69.23%	
**Identification with biological sex**				*χ*^*2*^ (1, *N* = 4936) = 13.23, *p* < .001
Yes	4908	6.61 (5.65), 0-27	24.04%	
No	28	9.18 (6.21), 0-19	53.57%	
**Civil status**				*χ*^*2*^ (1, *N* = 4936) = 142.25, *p* < .001
Single or divorced	2337	7.82 (6.00), 0-27	31.88%	
Married or in a civil partnership	2599	5.56 (5.11), 0-27	17.31%	
**Ethnicity**				*χ*^*2*^ (1, *N* = 4936) = 8.39, *p =* .004
Native	4634	6.57 (5.65), 0-27	23.76%	
Non-native	302	7.49 (5.81), 0-26	31.13%	
**Employment status**				*χ*^*2*^ (1, *N* = 4936) = 142.49, *p* < .001
Employed	3780	5.99 (5.06), 0-27	20.19%	
Unemployed	1156	8.72 (6.91), 0-27	37.37%	
**Predominantly socially distanced**^**a**^				*χ*^*2*^ (1, *N* = 4936) = 44.25, *p* < .001
Yes	3892	7.00 (5.77), 0-27	26.31%	χ^2^ (1, N = 4936) = 44.25, p < .001
No	1044	5.24 (5.02), 0-27	16.38%	

^a^Predominantly (i.e., at least 10 out of 14 days) socially distanced from peers and public activity related to pandemic protocols

^b^Not applicable: Too few participants within subgroup to meaningfully provide prevalence estimates.

Individuals who reported to have predominantly (i.e., at least 10 out of 14 days) socially distanced themselves from peers and public activity related to pandemic protocols reported substantially higher levels of impairing symptoms of anxiety (21.08% vs. 13.51%) and depression (26.31% vs. 16.38%) as compared to their counterparts. Follow-up analyses were conducted to investigate plausible confounders for this association. However, this relationship remained robust (*p* < .001) even after controlling for the simultaneous impact of previous and existing levels of psychopathological symptoms (i.e., depression and anxiety), psychiatric diagnosis, in addition to central demographics including age, gender, living situation, employment status, civil status, and urban vs. rural area residency, further demonstrating that the length of quarantine, isolation and adherence to social distancing protocols were associated with greater depressive and anxious symptomatology.

### Predictors of the trajectory of anxiety symptoms

As mentioned, anxiety symptoms significantly decreased from T1 to T2. The results from the linear mixed-effects model containing the predictors of the trajectory of anxiety symptoms can be found in Table 3. Model 1 shows the association between the demographic and the initial levels of mechanistic variables in March (T1) with changes in anxiety symptoms from March (T1) to July (T2). Inspecting the interactions with time portrayed that being male was associated with less reduction in anxiety symptoms from T1 to T2, following a similar trend as depicted for depressive symptoms in Supplementary Figure 2B. Older age was associated with greater reduction in anxiety symptoms from T1 to T2, with a sharper decrease in anxiety symptoms per year of age increased (Supplementary Figure 2A). Education was unrelated to the trajectory of anxious symptomology. Higher levels of negative metacognitive beliefs at T1 was associated with less reduction in anxiety symptoms from T1 to T2 (Figure 2). Higher baseline (T1) levels of physical activity were further associated with a sharper decrease in anxiety symptoms from T1 to T2, as depicted in Supplementary Figure 1B. Greater use of maladaptive coping strategies at T1 was associated with a sharper decrease in anxiety symptoms from T1 to T2, portraying a similar pattern as depicted for depressive symptoms in Supplementary Figure 1A. Higher levels of perceived competence at T1 was associated with less reduction in anxiety symptoms from T1 to T2, as illustrated in Figure 1. Baseline (T1) levels of positive metacognitive beliefs were unrelated to changes in anxious symptomology from T1 to T2.

**Table 3:** Linear mixed-effects models encompassing the predictors of the trajectory of anxiety symptoms from T1 (March 2020) to T2 (July 2020). The interaction terms in Model 1 reveal the extent the initial levels of the predictors at T1 have an association with changes in symptoms of anxiety from T1 to T2. The interaction terms in Model 2 illustrate how changes in the predictors from T1 to T2 are associated with the trajectory of anxiety symptoms from T1 to T2

	**Model 1**				**Model 2**			
	**Estimate**	***SE***	***t***	***p***	**Estimate**	***SE***	***t***	***p***
	Fixed effects							
Intercept	4.328	0.217	19.99	< .001	4.058	0.320	12.68	< .001
Time^a^	-0.209	0.344	-0.61	.543	-1.407	0.362	-3.88	< .001
Age	-0.007	0.002	-3.01	.002	-0.004	0.003	-1.49	.136
Sex^b^	-0.614	0.074	-8.27	< .001	-0.687	0.097	-7.10	< .001
Education	-0.122	0.033	-3.68	< .001	-0.101	0.044	-2.32	.020
PosMet^c^ (T1)	-0.003	0.000	-5.99	< .001	-0.001	0.001	-1.68	.093
NegMet^d^ (T1)	0.006	0.000	12.52	< .001	0.005	0.001	7.50	< .001
Coping Strategies^e^ (T1)	0.249	0.003	76.61	< .001	0.231	0.005	46.09	< .001
PhysAct^f^ (T1)	0.001	0.023	0.06	.955	-0.007	0.034	-0.19	.848
PerceComp^g^ (T1)	-0.882	0.042	-20.96	< .001	-0.750	0.058	-13.01	< .001
Time X Age	-0.012	0.003	-3.43	< .001	0.003	0.003	0.80	.425
Time X Sex	0.473	0.114	4.16	< .001	0.621	0.110	5.67	< .001
Time X Education	-0.043	0.051	-0.84	.402	0.013	0.049	0.27	.785
Time X PosMet (T1)	0.000	0.001	0.69	.493	0.000	0.001	0.30	.766
Time X NegMet (T1)	0.002	0.001	3.40	< .001	-0.004	0.001	-4.78	< .001
Time X Coping Strategies (T1)	-0.069	0.005	-14.03	< .001	-0.195	0.006	-34.33	< .001
Time X PhysAct (T1)	-0.094	0.035	-2.66	.008	0.009	0.039	0.24	.807
Time X PerceComp (T1)	0.374	0.065	5.78	< .001	0.513	0.065	7.87	< .001
PosMet (T2)					-0.001	0.001	-1.75	.080
NegMet (T2)					-0.001	0.001	-1.85	.064
Coping Strategies (T2)					0.043	0.005	8.66	< .001
PhysAct (T2)					0.068	0.035	1.92	.054
PerceComp (T2)					-0.185	0.063	-2.95	.003
Time X PosMet (T2)					-0.001	0.001	-1.18	.239
Time X NegMet (T2)					0.005	0.001	6.02	< .001
Time X Coping Strategies (T2)					0.198	0.006	35.49	< .001
Time X PhysAct (T2)					-0.070	0.040	-1.74	.082
Time X PerceComp (T2)					-0.146	0.071	-2.05	.040
	Random effects							
Intercept variance	2.92				2.44			
Residual variance	5.79				4.36			
AIC		74390.70				46371.97		

^a^T1 (March-April 2020) = 0, T2 (June-July 2020) = 1; ^b^ Male = 1, Female = 0; ^c^ Positive metacognitive beliefs; ^d^ Negative metacognitive beliefs; ^e^ Maladaptive coping strategies (i.e., Cognitive-attentional syndrome); ^f^ Physical activity; ^g^ Perceived competence to cope with pandemic challenges

Model 1 (Table 3) further allows for the examination of the association across time between the baseline (T1) variables and anxiety symptoms at T2, which can be extracted through the addition of the estimate for the main effect of the baseline variable with the estimate for its interaction with time. There was a significant association across time between perceived competence and anxiety symptoms, with higher levels of perceived competence at T1 associated with lower levels of anxiety symptoms at T2 (estimate = -0.508), as depicted in Figure 1. Higher initial levels of physical activity (T1) were related to lower levels of anxiety symptoms at T2 (estimate = -0.093), as illustrated in Supplementary Figure 1B. Higher initial (T1) levels of negative metacognitive beliefs were associated with higher levels of anxiety symptoms at T2 (estimate = 0.008), as depicted in Figure 2. There was a positive association across time (estimate = 0.180) between March levels of unhelpful coping strategies (T1) and anxiety symptoms in July (T2), indicating higher levels of symptoms connected to reliance on such strategies, despite the sharper decrease in symptoms from T1 to T2 (i.e., portraying a similar pattern as depicted in Supplementary Figure 1A for depressive symptoms). Being male was associated with lower levels of anxiety symptoms at T2 (estimate = -0.141). Additionally, older age was associated with lower levels of anxiety symptoms at T2 (estimate = -0.019 decrease in symptoms per year). Thus, older aged individuals start lower (i.e., are impacted less severely by the pandemic), reveal a trajectory with a sharper decrease in anxiety symptoms (i.e., recover faster), in addition to end up with lower levels of symptoms well into the pandemic (T2), portrayed in detail in Supplementary Figure 2A.

In addition to baseline associations with the trajectory, changes in mechanistic processes over time and their associations with the trajectory of anxiety were investigated. The interactions with Time and the T2 variables in Model 2 (Table 3) illustrates how changes (i.e., increase from T1 to T2) in the theorized mechanistic processes are related to the trajectory of anxious symptomology. Increased deployment of maladaptive coping strategies from T1 to T2 and elevations in negative metacognitive beliefs from T1 to T2 were associated with less reduction (i.e., less improvement) in anxious symptoms from T1 to T2. For maladaptive coping strategies, this is portrayed in Figure 3 revealing candidate trajectories of anxiety for prevalent and common scores in maladaptive coping strategies reported between T1 and T2. As depicted (i.e., lightest grey line), individuals who greatly decreased their deployment of maladaptive coping strategies (e.g., from a score of 28.1 at T1 to 1.4 at T2), experienced sharp decreases in anxiety symptoms from T1 to T2 (Figure 3; left corner). However, those increasing the deployment of such maladaptive coping strategies (e.g., from a score of 4.2 at T1 to 25.3 at T2; black line) reported substantial increases in anxious symptomatology (Figure 3; right corner). Changes in physical activity, competence to deal with the pandemic, and positive metacognitive beliefs from T1 to T2 were all unrelated with the trajectory of anxious symptomology from T1 to T2 at the pre-specified inference criteria when controlling for demographic characteristics and the initial (i.e., T1) levels of these variables.

### Predictors of the trajectory of depressive symptoms

As mentioned, there was a significant reduction in depressive symptoms from T1 to T2. Presented in Table 4, the interaction terms in Model 1 reveal the extent that demographic variables and the initial levels of the mechanistic variables in March (T1) were associated with changes in depressive symptoms from March (T1) to July (T2). Inspecting the interactions with time portrays that being male was associated with less reduction in depressive symptoms from T1 to T2 (Supplementary Figure 2B). Age and education were not significantly related to the trajectory of depressive symptoms when controlling for all other variables. Higher use of maladaptive coping strategies at T1 was associated with greater reduction depressive symptomatology from T1 to T2 (Supplementary Figure 1A). The levels positive metacognitive beliefs, negative metacognitive beliefs, perceived competence, and physical activity at T1 were unrelated to depressive symptoms at T2 at the pre-specified significance criteria. Model 1 further reveals the association across time between the baseline (T1) variables and depressive symptoms at T2, which can be extracted through the addition of the estimate for the main effect of the baseline variable with the estimate for its interaction with time. There was a positive association across time (estimate = 0.211) between maladaptive coping strategies at T1 with depressive symptoms at T2, indicating higher levels of symptoms for those relying on such strategies despite their sharper decrease in symptoms from T1 to T2, as depicted in Supplementary Figure 1A. Additionally, males were associated with lower levels of depressive symptoms at T2 (estimate = -0.205), portrayed in Supplementary Figure 2B.

**Table 4:** Linear mixed-effects models encompassing the predictors of the trajectory of depressive symptomology from T1 (March 2020) to T2 (July 2020). The interaction terms in Model 1 reveal the extent the initial levels of the predictors at T1 have an association with changes in depressive symptoms from T1 to T2. The interaction terms in Model 2 illustrates how changes in the predictors from T1 to T2 are associated with the trajectory of depressive symptoms from T1 to T2

	**Model 1**				**Model 2**			
	**Estimate**	***SE***	***t***	***p***	**Estimate**	***SE***	***t***	***p***
	Fixed effects							
Intercept	8.486	0.292	29.08	< .001	7.630	0.441	17.29	< .001
Time^a^	-0.681	0.417	-1.63	.102	-1.553	0.441	-3.52	< .001
Age	-0.037	0.003	-11.60	< .001	-0.028	0.004	-6.90	< .001
Sex^b^	-0.696	0.100	-6.96	< .001	-0.627	0.133	-4.70	< .001
Education	-0.438	0.045	-9.84	< .001	-0.429	0.060	-7.15	< .001
PosMet^c^ (T1)	-0.006	0.001	-9.19	< .001	-0.004	0.001	-4.20	< .001
NegMet^d^ (T1)	0.011	0.001	17.39	< .001	0.009	0.001	9.26	< .001
Coping Strategies^e^ (T1)	0.264	0.004	60.34	< .001	0.223	0.007	32.31	< .001
PhysAct^f^ (T1)	-0.348	0.032	-11.00	< .001	-0.336	0.047	-7.12	< .001
PerceComp^g^ (T1)	-0.636	0.057	-11.21	< .001	-0.598	0.080	-7.52	< .001
Time X Age	0.008	0.004	1.99	.047	0.023	0.004	5.59	< .001
Time X Sex	0.491	0.138	3.56	< .001	0.618	0.133	4.64	< .001
Time X Education	-0.009	0.062	-0.15	.881	0.072	0.060	1.20	.230
Time X PosMet (T1)	0.002	0.001	2.31	.021	0.003	0.001	2.80	.005
Time X NegMet (T1)	0.000	0.001	0.14	.891	-0.007	0.001	-7.14	< .001
Time X Coping Strategies (T1)	-0.053	0.006	-8.92	< .001	-0.182	0.007	-26.31	< .001
Time X PhysAct (T1)	-0.072	0.042	-1.69	.091	0.125	0.047	2.66	.008
Time X PerceComp (T1)	0.164	0.078	2.09	.037	0.403	0.079	5.07	< .001
PosMet (T2)					0.000	0.001	-0.20	.843
NegMet (T2)					-0.001	0.001	-1.00	.318
Coping Strategies (T2)					0.087	0.007	12.76	< .001
PhysAct (T2)					0.097	0.049	1.99	.005
PerceComp (T2)					-0.078	0.086	-0.91	.363
Time X PosMet (T2)					-0.003	0.001	-3.34	.001
Time X NegMet (T2)					0.006	0.001	6.59	< .001
Time X Coping Strategies (T2)					0.201	0.007	29.62	< .001
Time X PhysAct (T2)					-0.275	0.049	-5.65	< .001
Time X PerceComp (T2)					-0.273	0.086	-3.16	.002
	Random effects							
Intercept variance	7.75				6.47			
Residual variance	8.07				6.45			
AIC		82539.09				51930.84		

^a^T1 (March-April 2020) = 0, T2 (June-July 2020) = 1; ^b^ Male = 1, Female = 0; ^c^ Positive metacognitive beliefs; ^d^ Negative metacognitive beliefs; ^e^ Maladaptive coping strategies (i.e., Cognitive-attentional syndrome); ^f^ Physical activity; ^g^ Perceived competence to cope with pandemic challenges.

Model 2 in Table 4 presents the details concerning how change (i.e., increase from T1 to T2) in the mechanistic processes are related to the trajectory of depressive symptomology. Increases in deployment of maladaptive coping strategies and negative metacognitive beliefs from T1 to T2 was associated with less reduction (i.e., less improvement) in depressive symptoms from T1 to T2, portraying a similar pattern as depicted for anxiety symptoms in Figure 3. Increasing physical activity from T1 to T2 (Figure 4) as well as increases in perceived competence from T1 to T2 (Supplementary Figure 3A) was associated with greater reduction of depressive symptoms from T1 to T2. Increases in positive metacognitive beliefs from T1 to T2 was associated with greater reduction of depressive symptoms (Supplementary Figure 3B).

## Discussion

### Prevalence of anxiety and depressive symptoms

In contrasting the onset of the pandemic (i.e., T1) with four months into the pandemic period (i.e., T2), fewer participants reported impairing levels of depressive and anxiety symptoms at T2, where the social distancing protocols were substantially lightened in severity as compared to T1. All previously identified vulnerable subgroups at the onset of the pandemic maintained their relative positions four months into the pandemic, suggesting the continued proneness of these identified groups over time through the pandemic period. These results strengthen the notion concerning the additional mental health toll experienced by females, those who are single or divorced, unemployed, individuals belonging to ethnic and sexual minorities, and those residing alone during the pandemic (e.g., Salari et al., 2020; Santabárbara et al., 2021), highlighting the need for deployment of community-level strategies that may reduce the burden of the pandemic and its protocols for these subgroups. The prolonged and unmodified suffering of these subgroups under the pandemic further points to the necessity for implementation of novel and alternative strategies aimed at reducing detrimental mental health symptoms. Given the high prevalence of adverse symptoms levels within these subgroups, wide-dissemination strategies including the utilization of self-help interventions tailored toward pandemic-specific concerns may be warranted to reach out to as many individuals as possible. Such strategies have previously been found to be fruitful in alleviating symptoms of anxiety and depression in non-pandemic settings (e.g., Morgan et al., 2017). Other vulnerable subgroups than those identified in the present study have also been noted in the literature, such as adolescents and parents (e.g., Commodari & La Rosa, 2020; Johnson et al., 2021), collectively highlighting the necessity to direct increased efforts toward groups that are more skewedly affected by the pandemic.

Additionally, individuals who had predominantly socially distanced themselves from peers and public activity as related to pandemic protocols reported higher levels of detrimental symptoms of anxiety and depression. This association was robust when controlling for the simultaneous impact of a large number of plausible covariates, suggesting there may be deleterious associations between the pandemic mitigation protocols and mental health symptoms. Prospective, case-control studies, and meta-analyses in the pandemic literature have yielded mixed findings concerning the association between social distancing protocols and mental health symptoms, with some identifying pernicious associations (e.g., Ettman et al., 2020; Wang et al., 2021a; Wu et al., 2020) and other studies no such substantial associations (e.g., Castaldelli-Maia et al., 2021). The study at hand identified decreases in these symptoms between a period of strict mitigation protocols as compared to a period where these protocols were predominantly lightened in severity in a sample where the same participants experienced both variations of these regulatory strategies, serving as their own controls, while further controlling for plausible confounders including current and pre-existing symptoms levels, psychiatric diagnosis, and key demographic factors. These findings are further consistent with a longitudinal study in Norway portraying that symptom severity increase and decreases in accordance with strictness of pandemic mitigation protocols (Norwegian Institute of Public Health, 2020), in addition to a large-scale longitudinal study of 200 000 individuals in Northern and Western European countries which are more similar to the present sample, revealing notable impacts of mitigation protocols on the mental health of the population (Varga et al., 2021).

Taken together, the detrimental association of symptoms with mitigation protocols and the additional and continued heightened scores of identified in-risk subgroups suggest that important interventive efforts in the present pandemic, and preventive efforts for future periods of infectious disease, must be taken to protect individuals against adverse symptomatology. This highlights the necessity of simultaneously keeping mental health in mind while these important viral mitigation protocols are in place, focusing on interventions that may alleviate detrimental symptoms. Interventions of additional utility at the population level could involve the use of social bubbles during periods with visitation prohibition, particularly for individuals residing alone and those who are single or divorced. This recommendation is further in line with the associations identified between loneliness, social isolation, and social support with symptoms of anxiety and depression during the present pandemic (e.g., Hoffart et al., 2020; Hoffart et al., 2021; Santabárbara et al., 2021),

### Trajectorial changes in anxiety and depression related to stable factors and baseline levels of predictors

Several demographic variables were differentially associated with the trajectorial changes within symptoms of anxiety and depression. Meta-analyses of cross-sectional studies have associated younger aged adults with detrimental symptoms of depression and anxiety during the onset of the pandemic (e.g., Salari et al., 2020; Wu et al., 2020). The present article adds to the literature by illustrating that young adults not only have more adverse immediate responses to the pandemic, but further a) reveal the least favorable trajectory of improvement in anxiety symptoms, demonstrating slower rates of recovery from detrimental symptoms, in addition to b) continuing to experience higher symptoms well into the pandemic. This finding highlights the substantial maintained risk of younger adults to experience anxiety symptoms during periods of infectious disease. Given the simultaneous exposure to the pandemic, the greater resiliency of older adults is an important area for future studies to investigate, with mechanisms related to these lower symptom levels remaining to be identified and advantageous in the study of pandemic resiliency. A possible explanation of the age-related differences in trajectories and levels of anxiety symptoms may include the differential impact of mitigations protocols on the social daily life of different age groups. Although all age groups are impacted by drastic changes to daily life, higher age has been found to be associated with declines in social activities including time spent with others (e.g., Marcum, 2013), thus highlighting that younger adults may be experiencing greater perturbations to their social daily life. These larger perturbations, in addition to being pernicious by themselves, may elicit more frequent use of maladaptive coping responses, including worry and rumination, increases in which the present study found to be detrimentally associated with anxiety symptoms. It is worth noting although the association between age and the trajectory of depressive symptoms (*p* = .047) did not surpass the present studies significance criteria, the trajectorial trend as associated with age was opposite for depression compared to anxiety. In contrast to anxiety, older age was related to slower rates of improvement from depressive symptoms, suggesting that young adults experience quicker recovery rates from depressive symptoms, while older aged individuals recover faster from anxiety. These preliminary tendencies are imperative to investigate in greater depth in future studies, further attesting to the suggested importance of studying the trajectories of depression and anxiety separately (e.g., Fancourt et al., 2021). Concerning sex, although females reported higher initial levels of depressive and anxiety symptoms, their trajectory was characterized by a sharper decrease in both symptom domains. These results are consistent with findings from Fancourt et al. (2021). Thus, it seems that the pandemic protocols have more adverse associations for females, as females both demonstrate a heightened initial response to these protocols in addition to faster recovery following their discontinuation. This is consistent with previous studies (e.g., Flaherty & Richman, 1989) highlighting that females to a greater extent are impacted by their social support networks than males, with the discontinuation of protocols allowing individuals to re-engage with their social support networks thus allowing possibly allowing females to recover faster from adverse symptoms. Still, despite their greater rate of improvement, females continued to experience higher levels of anxiety and depressive symptoms at T2, consistent with other cross-sectional portrayals of gender disparities during the pandemic (e.g., Salari et al., 2020).

When examining the association between the baseline (T1) levels of mechanistic variables and the trajectories of interest, several important differences emerged with respect to these variables’ associations with the course of anxiety and depressive symptoms, respectively. Although baseline levels of negative metacognitive beliefs were unrelated to change in depressive symptoms, their higher levels at baseline were associated with less reduction in anxiety symptoms across time. Similarly, baseline levels of physical activity were differentially related to depression and anxiety in a distinct and specific pattern. Although there was a positive simultaneous association between physical activity at T1 and depression at T1, no such simultaneous relationship was found between physical activity and anxiety symptoms. However, higher initial levels of physical activity was associated with accelerated recovery from adverse anxiety symptoms, indicating that physical activity is related to long-term advantages rather than being associated with simultaneous alleviations for anxiety as opposed to depressive symptoms. This pattern between physical activity and anxiety is depicted in detail in Supplementary Figure 1B. This finding is important, as relying solely on cross-sectional patterns would neglect an association between physical activity and anxiety during the pandemic (e.g., Ebrahimi et al., 2021a). The present findings of long-term associations between physical activity and anxiety suggests that facilitating and aiding individuals in maintaining their physical activity levels represents a promising strategy of aid in the alleviation of anxiety symptoms over time, which may be fostered by introducing community-level strategies mindful of infectious transmission aimed at keeping individuals active during pandemic periods, and by clinicians in psycho-educative settings informing clients about the delayed rather than immediate associations of physical activity with anxious symptomatology. These findings fill important gaps in the literature concerning the differential association of variables with the unique trajectories of depression and anxiety as called for by several scholars (e.g., Fancourt et al., 2021; Riehm et al., 2021). Additionally, higher initial levels of reliance on maladaptive coping strategies were associated with greater reduction of both anxiety and depressive symptoms, while higher baseline levels of perceived competence to cope with the pandemic revealed this pattern only for anxiety. These findings seem to reflect cases of regression to the mean, given that those with higher initial values on maladaptive strategies and lower perceptions of competence to deal with the pandemic crisis also report higher levels of symptoms, as portrayed in Figure 1 and Supplementary Figure 1A. Both the aforementioned groups (i.e., individuals lower in perceived competence, and those high in use of maladaptive strategies at baseline) further report higher symptoms levels at T2, providing further support for this interpretation.

### Trajectorial changes in anxiety and depression as related to changes over time in mechanistic variables

Importantly, mechanisms of relevance were investigated by inspecting how change in mechanistic processes across time (i.e., from the onset (T1) to the later stages (T2) of the pandemic) were associated with the trajectory of anxiety and depressive symptoms. Both the increased reliance on maladaptive coping strategies from T1 to T2 and increase in negative metacognitive beliefs from T1 to T2 were related to less improvement in depressive and anxious symptoms across time. These findings provide support for metacognitive theory of psychopathology (Wells, 2009), which highlights how depression and anxiety may be prolonged through reliance on preservative thinking styles and maladaptive coping strategies driven by metacognitive beliefs. As the pandemic has amplified the reliance on preservative thinking styles (i.e., worry and rumination) in addition to maladaptive coping strategies (e.g., alcohol consumption) having increased during the pandemic (e.g., Capasso et al., 2021; Cheng et al., 2021; Rodriguez et al., 2020; Taylor et al., 2021; Varga et al., 2021), the strong associations between these mechanisms and the trajectory of psychopathological symptoms are of particular concern. Therapeutic modalities centered around fostering changes in these mechanistic processes have previously yielded reduced symptom severity in randomized trials (e.g., Hanrahan et al., 2013; Watkins, 2015), including metacognitive therapy which specifically aims at reducing metacognitions and maladaptive coping strategies (e.g., Hjemdal et al., 2019; Johnson et al., 2017). Figure 3 further portrays how even small changes in maladaptive coping strategies have substantial impact on the trajectory of anxious and depressive symptomatology, even after controlling for the effect of all other mechanisms and demographic variables in the model. Manipulations of such processes is therefore important and is likely to foster similarly beneficial repercussions in pandemic settings, although the latter assertion warrants investigation in forthcoming studies.

Increases in physical activity levels and perceptions of greater competence to cope with the pandemic from T1 to T2 were both significantly associated with trajectory of depression, portraying faster improvement from adverse depressive symptoms. These trends followed a similar pattern but were insignificant in relation to the trajectory of anxiety. A plausible explanation for the stronger association between perceived competence for depression as compared to anxiety may include the greater impact increases in such a mechanism has for depression, as the depression is more strongly related to hopelessness and helplessness than anxiety (e.g., American Psychiatric Association, 2013; Schroder & Ollis, 2013). As the pandemic presents a multitude of novel situations of which individuals are uncertain how to cope with, the use of informative campaigns providing practical guidance on typical challenges that will arise during such periods may present a feasible strategy towards amelioration of depressive symptoms in pandemic settings. In clinical settings, several empirically grounded methods exist upon which problem-solving and skills training approaches may be used to foster individuals’ beliefs in their ability to cope with challenges (e.g., Cuijpers et al., 2018). Regarding physical activity, these findings yet again demonstrate the importance of taking measures that enable individuals to engage in physical exercise during pandemics, which overall reveal distinct patterns of association with depressive and anxious symptoms, with the associated ameliorations for anxiety revealing the presence of delayed impact of physical exercise, while alleviation in depressive symptomatology reveals a greater momentaneous relationship with physical exercise. Improvements in depressive symptoms are thus associated both with the simultaneous exertion as well as dose-increases in exercise over time in exercise.

Increases in positive metacognitive beliefs from T1 to T2 was associated with greater reduction in depressive symptoms across time, but this change in metacognitive activity was unrelated to anxiety. Positive metacognitions include thoughts such as “analyzing my problems will help me identify their solutions”, commonly associated with detrimental symptoms of depression and anxiety in clinical populations (Wells, 2009). These associations were however not replicated in the present pandemic community sample. A possible explanation for this discrepancy may be that one of the items measuring positive metacognitive beliefs (i.e., “focusing on possible threat can keep me safe”) may behave differently in pandemic settings, where heightened focus on threatful stimuli is a natural response in the general population without necessarily reflecting a pathological mechanism, such as superfluous threat monitoring. Thus, it may be possible that this introduces bias into the common conceptualization of positive metacognitive beliefs, highlighting that the reader should interpret this association cautiously.

### Strengths and limitations

The present study has some notable strengths and limitations. Although the preponderance of the participants were randomly obtained, conducting this procedure online may have favored particular groups of individuals above others (e.g., younger participants versus elderly), which serve as a limitation of this study. Major steps were however taken to reduce this possible bias through the recruitment of participants across a variety of platforms which were more accessible to the elderly population. Physical activity was measured unidimensionally and the study could have been further strengthened by specifying the type of activity and conducting operationalization with respect to the WHO-criteria of physical activity. A further limitation includes the use of self-reported symptom assessments rather than clinician-administered interviews, precluding evaluation of diagnostic status. Another limitation of the present study concerns the presence of overrepresented subgroups, including females and individuals with higher education). Still, sensitivity analyses on fully randomly selected proportion of participants as well as adjusted set and weighted set of participants in the same sample revealed identical results as the full sample, which is not surprising giving that the underrepresented subgroups (e.g., males) consist of a substantially large number of individuals (e.g., *n =* 2184 at T1 and 1010 at T2). The study further holds several strengths, including its stringent designed mapped against modifications of pandemic protocols, in addition to the use of maximum likelihood, the state of art estimation for missing values. The investigation of mechanistic processes subjectable to change rather than predominant emphasis on stable demographics includes a major strength of the present study, presenting insights of increased utility in clinical settings. Finally, the identification of mechanisms associated fluctuations in depressive and anxious symptomatology may above alleviation of such deleterious mental health states provide aid in minimizing other detrimental covariates associated with such symptoms, including panic buying (e.g., Asmundson & Taylor, 2020), risk of COVID-19 infection (e.g., Wang et al., 2021b), in addition to aiding with minimization of strategies (e.g., alcohol consumption) tied with efforts to cope with such symptomatology which are further associated with lower adherence to mitigation protocols and proliferation of the virus (e.g., Ebrahimi et al., 2021b).

### Concluding remarks

Mechanistic processes are divergently related to the trajectory of anxious and depressive symptomatology during the pandemic. Knowledge concerning the distinct patterns of associations between mechanisms and different domains of psychopathology are imperative toward informing preventive and interventive efforts in ameliorating the adverse mental health symptoms that have surged during the pandemic. Self-help interventions centered around the highlighted mechanistic processes in the present article, including the reduction of maladaptive coping strategies and negative metacognitions may present a potent strategy in reducing the symptom burden of both depression and anxiety accompanying pandemics, with the additional possibility of reaching a wide range of individuals. Physical activity and increasing knowledge concerning how to best cope with pandemic challenges may further present relevant interventive targets, although these mechanisms were differentially associated with the trajectories of anxious and depressive symptoms.

## Supplementary Information

ESM 1 (PDF 104 kb)

ESM 2 (PDF 5 kb)

ESM 3 (PDF 8 kb)

ESM 4 (PDF 10 kb)
