# Supplementary material for: Mechanisms associated with the trajectory of depressive and anxiety symptoms: A linear mixed-effects model during the COVID-19 Pandemic
Source: Curr Psychol. 2022 Feb 4:1–18. Online ahead of print. doi: 10.1007/s12144-022-02732-9 (PMC8816311; doi:10.1007/s12144-022-02732-9)
Supplement: Supplementary file 1 — (PDF 104 kb) [file 12144_2022_2732_MOESM1_ESM.pdf]

Supplementary Table S1. All nationally initiated non-pharmacological interventions during the COVID-19 pandemic in Norway actively in place during the first wave of data collection (T1; between March 31 to April 7, 2020). NPIs were identical across all regions and throughout the measurement period. No new information was given about modifications of NPIs during the measurement period, controlling for expectation effects.

**Non-pharmacological intervention (NPI)**	**Duration of NPI**	**Modification or novel information about modification of NPI provided during the measurement period**
1. Individuals who have been in contact with anyone who has been infected by the disease are quarantined for 14 days following initial contact with the infected person.	Two weeks prior to and during the measurement period.	No
2. Anyone suspecting symptoms of the coronavirus or is confirmed to have the virus must be in isolation.	Two weeks prior to and during the measurement period.	No
3. Social and physical distancing: individuals are disallowed from being in groups with more than five people and must maintain at least two meters distance from others.	Two weeks prior to and during the measurement period.	No
4. Closing schools, kindergartens, and universities.	Two weeks prior to and during the measurement period.	No
5. Closing of all businesses in the catering, food, and beverage industry. The exception of the rule involves eateries that may facilitate visitors to have at least a one-meter distance from each other.	Two weeks prior to and during the measurement period.	No
6. Closure of all additional businesses with increased risk of infectious spread. This includes any business involving human contact, with the exception of essential stores (e.g., grocery stores, pharmacies).	Two weeks prior to and during the measurement period.	No
7. Individuals returning to Norway receive an automatic quarantine duration of 14 days.	Two weeks prior to and during the measurement period.	No
8. Cancellation of cultural events (e.g., concerts), closing of gyms, and physical work-out centers.	Two weeks prior to and during the measurement period.	No
9. Health personnel disallowed from leaving the country.	Two weeks prior to and during the measurement period.	No
10. All hospitals and health institutions must introduce access control and stop regular visitation routines.	Two weeks prior to and during the measurement period.	No
11. Ban on traveling to and staying overnight at one’s leisure property outside the individuals residing municipality.	Two weeks prior to and during the measurement period.	No
12. Border control: The borders are closed with regards to visitors from other countries.	Two weeks prior to and during the measurement period.	No

Supplementary Table S2. All nationally initiated non-pharmacological interventions during the COVID-19 pandemic in Norway actively in place during the second wave of data collection (T2; between June 22 to July 13, 2020). NPIs were identical across all regions and throughout the measurement period. No new information was given about modifications of NPIs during the measurement period, controlling for expectation effects. As with T1, all NPIs were stable and unchanged for the weeks prior to and during data collection.

**Non-pharmacological intervention (NPI)**	**Duration of NPI**	**Specific modification conducted since onset of the pandemic (T1 measurement)**	**Modification or novel information about modification of NPI provided during the measurement period**
1. Individuals who have been in contact with anyone who has been infected by the disease are quarantined for 10 days following initial contact with the infected person.	Since the onset of the pandemic protocols in Norway (March 12, 2020).	Quarantine period reduced from 14 to 10 days. *Modified May 7, 2020.*	No
2. Anyone suspecting symptoms of the coronavirus or is confirmed to have the virus must be in isolation.	Since the onset of the pandemic protocols in Norway (March 12, 2020).	*Unmodified.*	No
3. Social and physical distancing: individuals are disallowed from being in groups with more than twenty people and must maintain at least a one-meter distance from others.	Since the onset of the pandemic protocols in Norway (March 12, 2020).	Distance reduced from two meters to one meter. (*Modified May 7, 2020)*	No
4. Universities and colleges are closed (Elementary and high school have re-opened)	Since the onset of the pandemic protocols in Norway (March 12, 2020).	Elementary and high schools are re-opened. (*Modified May 7, 2020)*	No
5. Individuals visiting or returning to Norway receive an automatic quarantine duration of 10 days.	Since the onset of the pandemic protocols in Norway (March 12, 2020).	Quarantine period reduced from 14 to 10 days. *(Modified May 7, 2020)*	No
6. Public events must not exceed more than 200 individuals. In this case, they may be allowed if events can maintain the one-meter distance rule and meet the requirement of infection control protocols.	Introduced May 7, 2020.	Public events re-allowed given specific conditions. *(Introduced May 7, 2020)*	No
7. Re-opening of direct contact health service providers (e.g., psychologists and physiotherapists) provided they meet the requirement of infection control protocols.	Introduced April 20, 2020.	Re-opening. *(Introduced May 7, 2020)*	No
8. Re-opening of one-to-one contact services (e.g., hair salons), gyms, and the catering and beverage industry may re-open provided they meet the requirement of infection control protocols (as well as the maintenance of a one-meter distance for gyms and the catering and beverage industry).	Introduced April 27, 2020 (contact services), May 7, 2020 (gyms) and June 1, 2020 (catering and beverage industry).	Re-opening. *(Introduced respectively: 27 April, May 7, and June 1, 2020)*	No
9. All hospitals and health institutions must introduce access control and stop regular visitation routines.	Since the onset of the pandemic protocols in Norway (March 12, 2020)	*Unmodified.*	No
