# Supplementary material for: Mechanisms associated with the trajectory of depressive and anxiety symptoms: A linear mixed-effects model during the COVID-19 Pandemic
Source: Curr Psychol. 2022 Feb 4:1–18. Online ahead of print. doi: 10.1007/s12144-022-02732-9 (PMC8816311; doi:10.1007/s12144-022-02732-9)

Supplementary Figure 1A. Trajectory of Depressive Symptoms predicted by baseline (T1) levels of Maladaptive Strategies

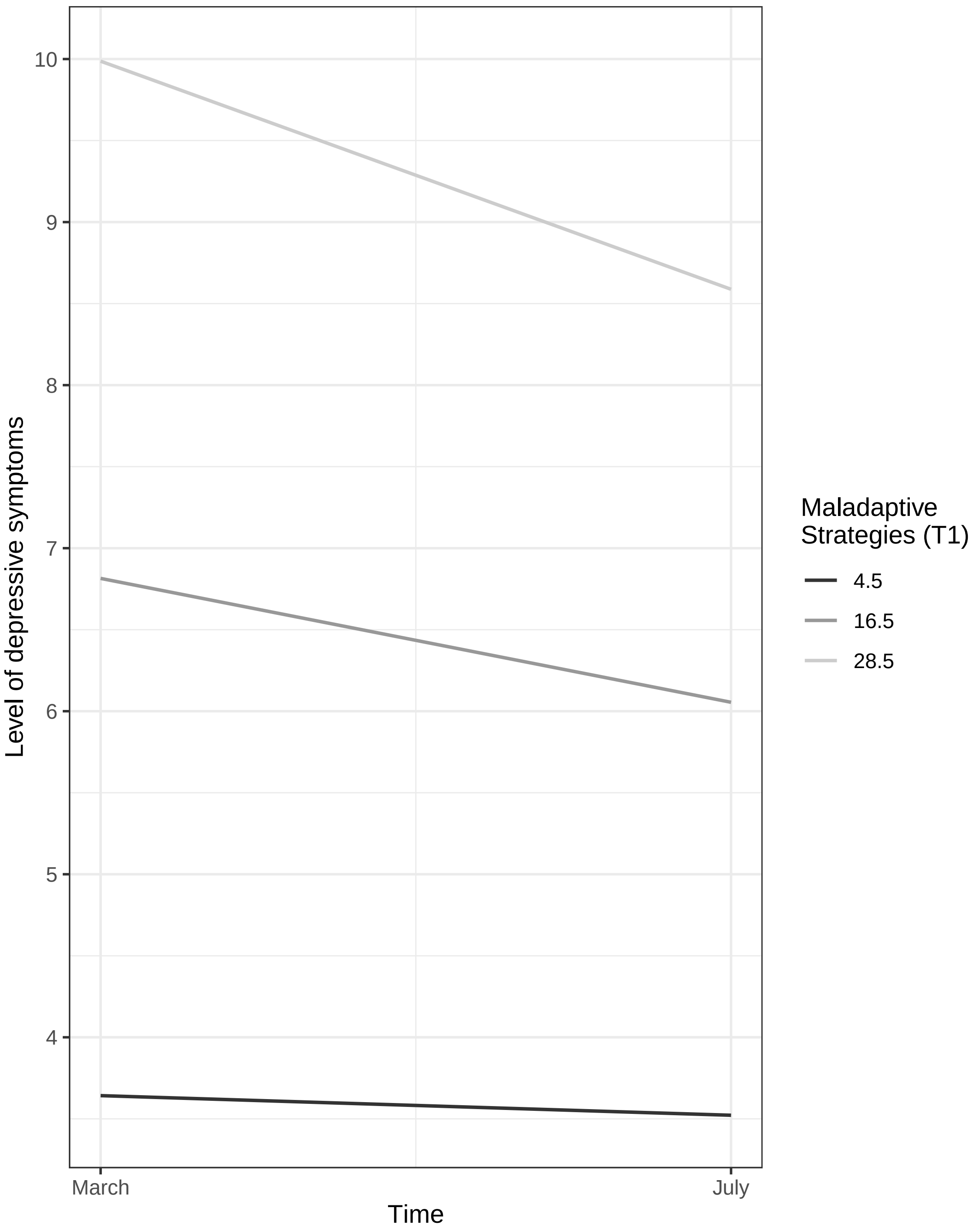

Supplementary Figure 1B. Trajectory of Anxiety predicted by baseline (T1) Physical Activity

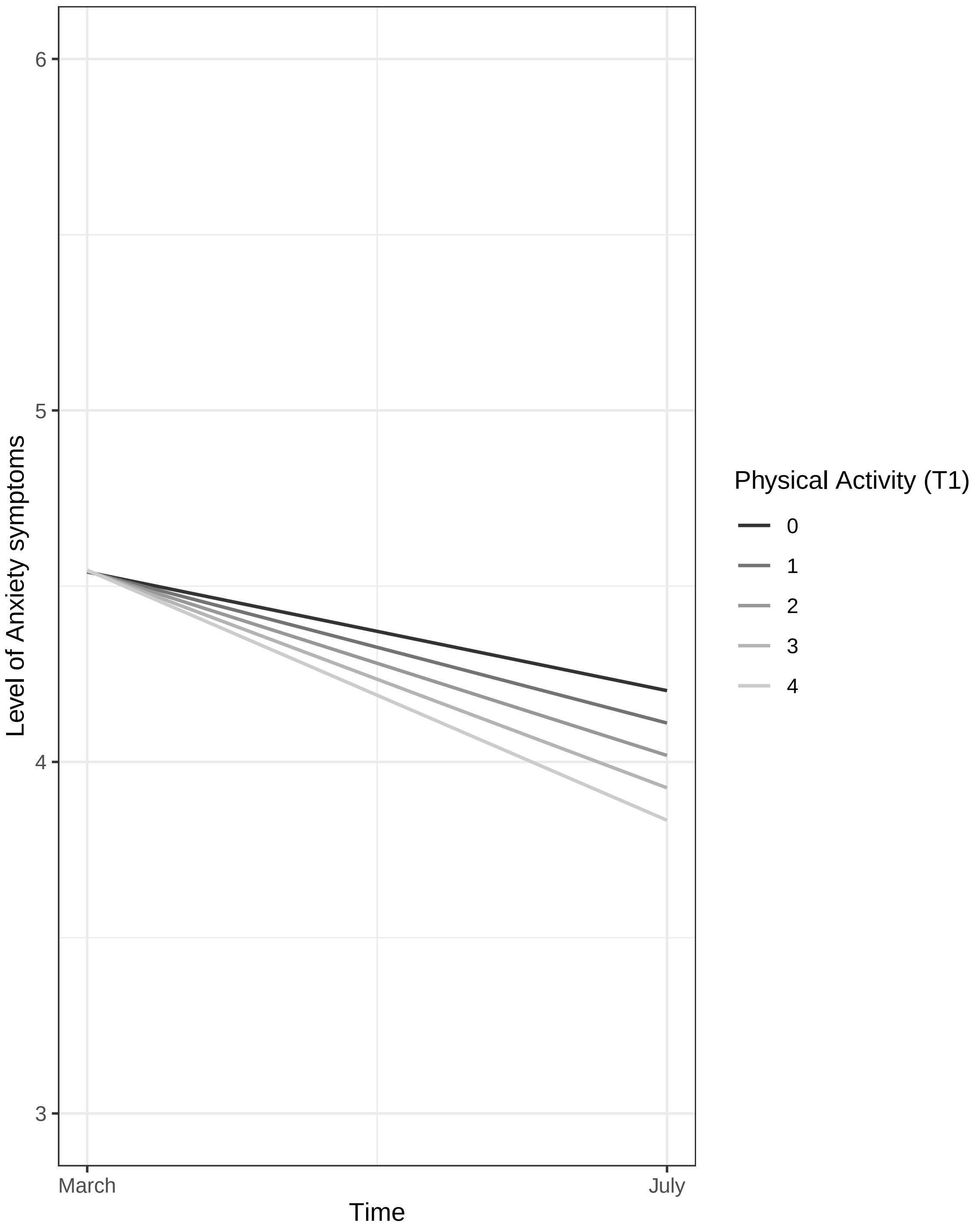

Supplement: Supplementary file 2 — (PDF 5 kb) [file 12144_2022_2732_MOESM2_ESM.pdf]
