# Supplementary material for: Mechanisms associated with the trajectory of depressive and anxiety symptoms: A linear mixed-effects model during the COVID-19 Pandemic
Source: Curr Psychol. 2022 Feb 4:1–18. Online ahead of print. doi: 10.1007/s12144-022-02732-9 (PMC8816311; doi:10.1007/s12144-022-02732-9)

Supplementary Figure 2A. Trajectory of Anxiety Symptoms predicted by Age

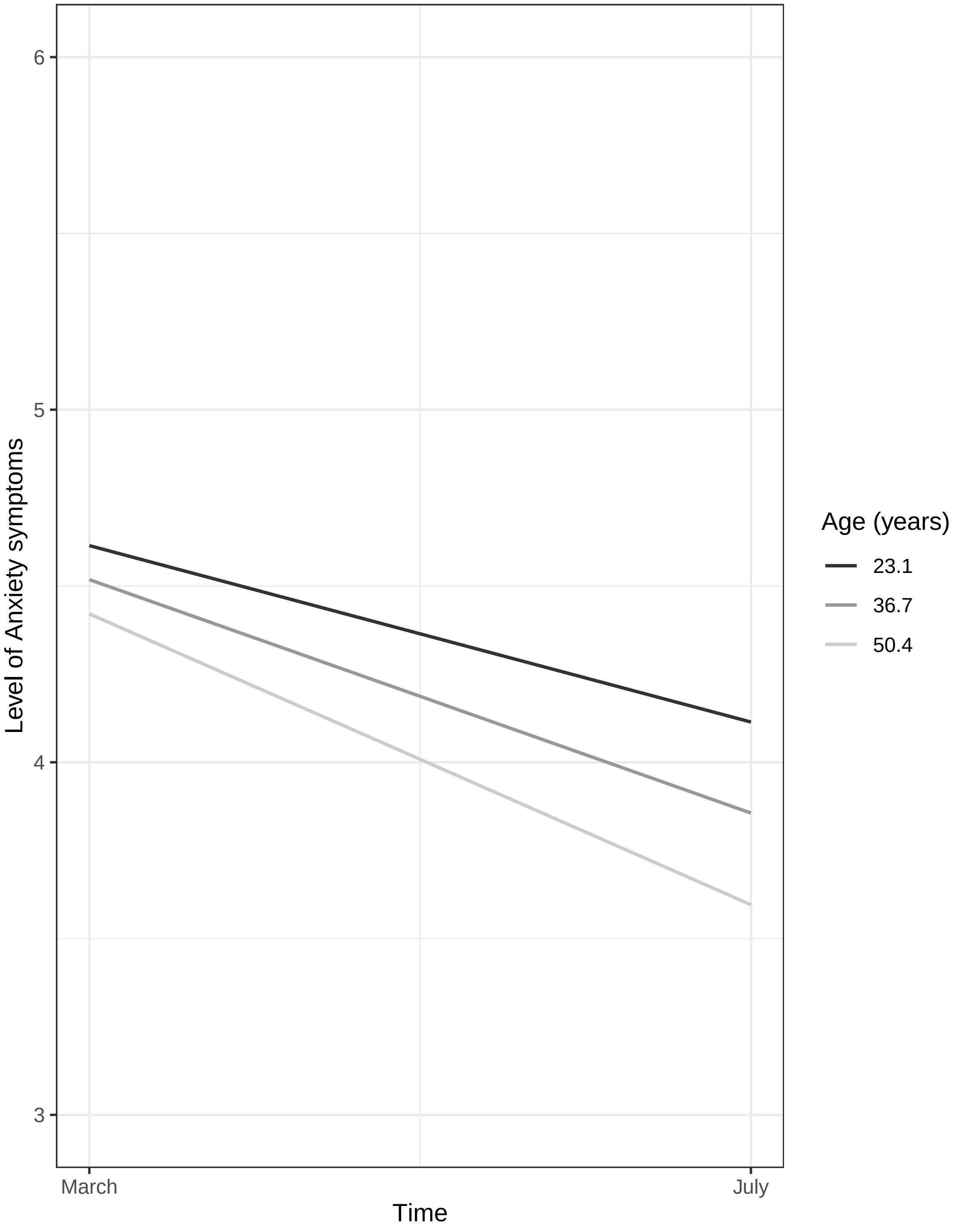

Supplementary Figure 2B. Trajectory of Depressive Symptoms predicted by Sex

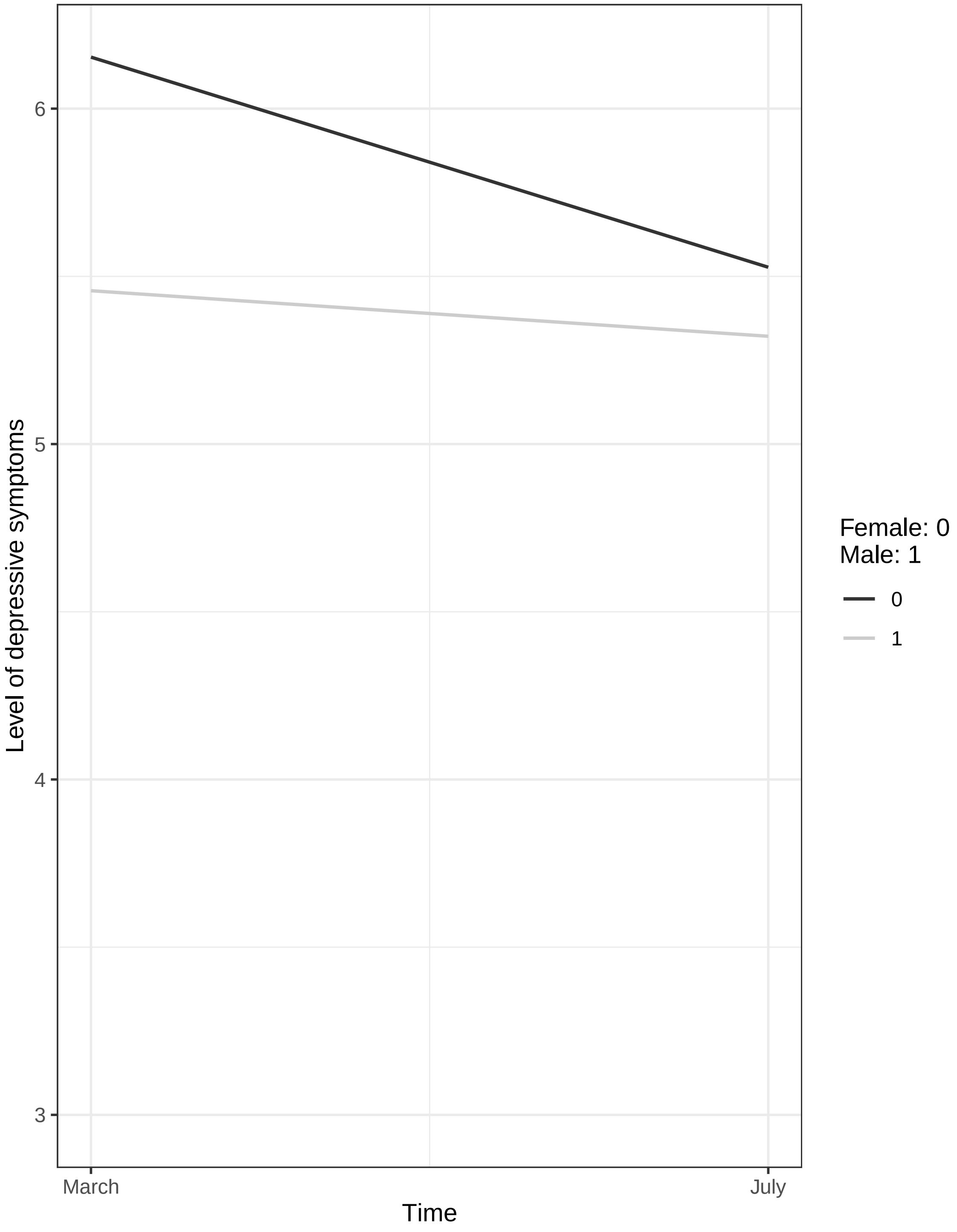

Supplement: Supplementary file 3 — (PDF 8 kb) [file 12144_2022_2732_MOESM3_ESM.pdf]
