# Supplementary material for: Mechanisms associated with the trajectory of depressive and anxiety symptoms: A linear mixed-effects model during the COVID-19 Pandemic
Source: Curr Psychol. 2022 Feb 4:1–18. Online ahead of print. doi: 10.1007/s12144-022-02732-9 (PMC8816311; doi:10.1007/s12144-022-02732-9)

Supplementary Figure 3A. Trajectory of Depression predicted by changes in Perceived Competence from T1 to T2

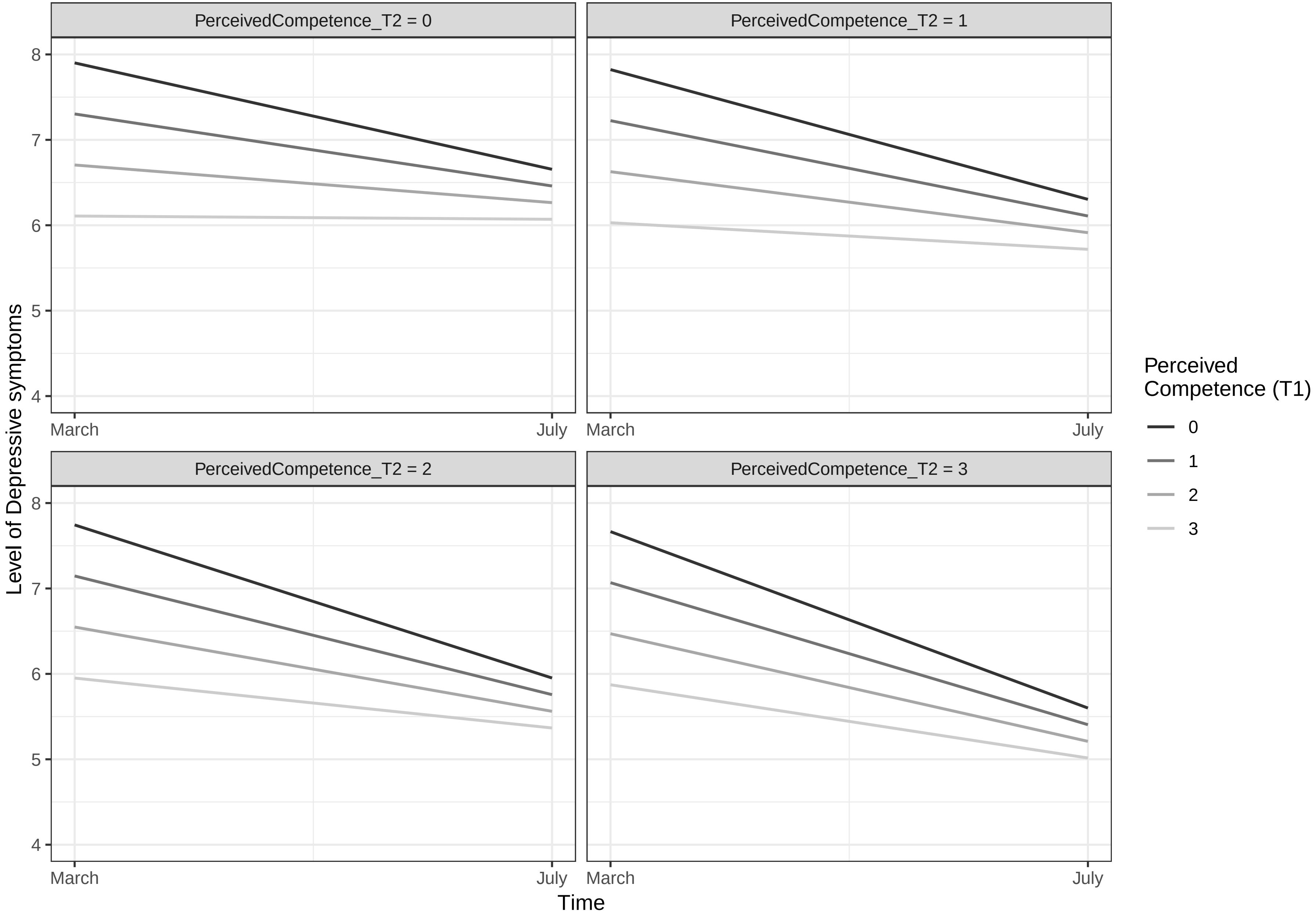

Supplementary Figure 3B. Trajectory of Depression predicted by changes in Positive Metacognitions from T1 to T2

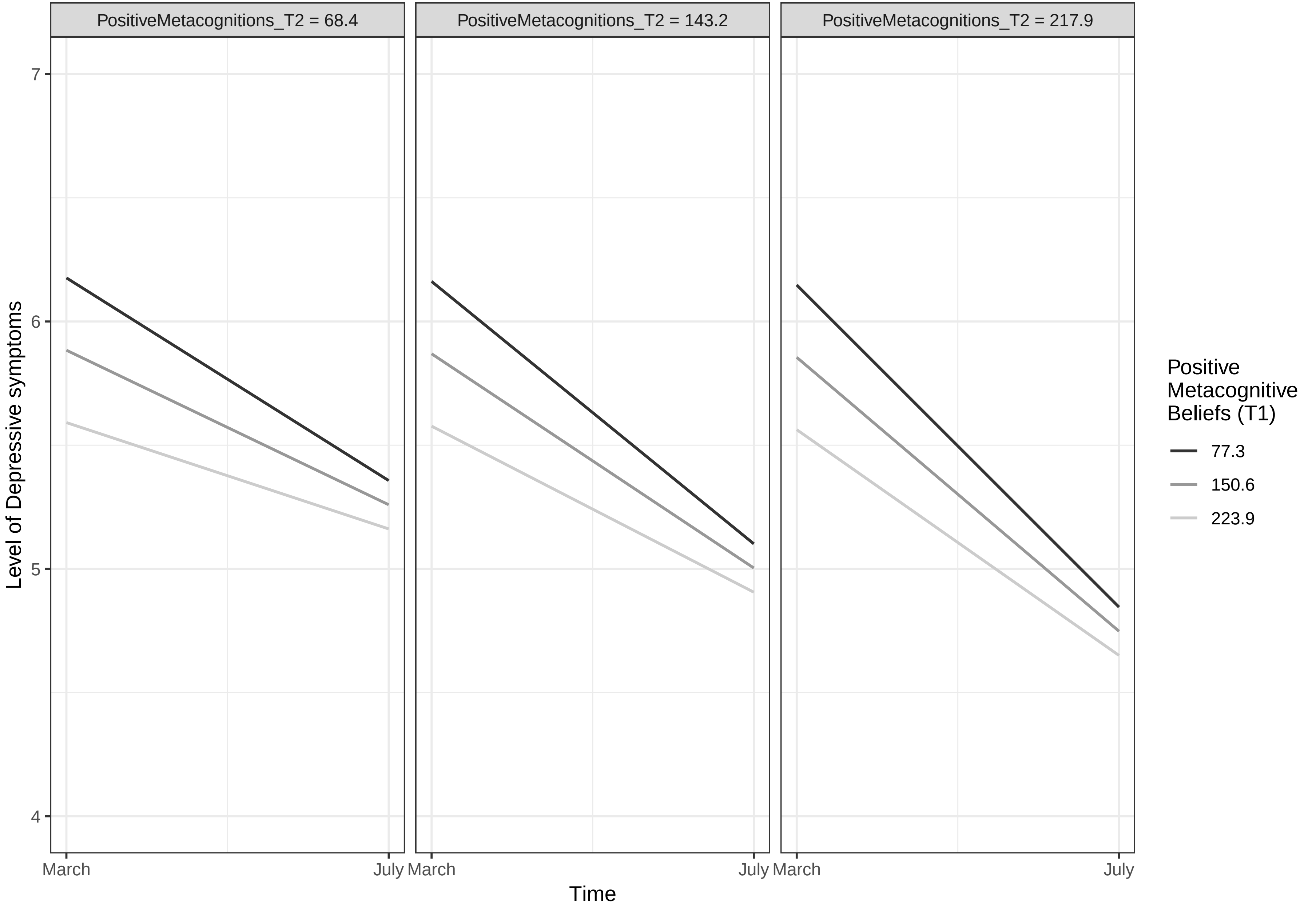

Supplement: Supplementary file 4 — (PDF 10 kb) [file 12144_2022_2732_MOESM4_ESM.pdf]
